# A Therapeutic Approach to Primary Hyperparathyroidism: A Collaborative Evaluation Between Endocrinology and Surgery

**DOI:** 10.7759/cureus.34157

**Published:** 2023-01-24

**Authors:** Andrew J Behnke, Daniel R Tershak, William F Abel, Adegbenga A Bankole

**Affiliations:** 1 Endocrinology, Diabetes and Metabolism, Virginia Tech Carilion School of Medicine, Roanoke, USA; 2 General Surgery, Virginia Tech Carilion School of Medicine, Roanoke, USA; 3 Internal Medicine, Carilion Clinic, Roanoke, USA; 4 Rheumatology, Virginia Tech Carilion School of Medicine, Roanoke, USA

**Keywords:** patient-centred care, quality improvement projects, head neck surgery, osteoporosis, recurrent nephrolithiasis, acute hypercalcemia, hyperparathyroid-induced hypercalcemia, adult primary hyperparathyroidism

## Abstract

Introduction

Primary hyperparathyroidism (PHPT) is a disorder in which one or more parathyroid glands overproduce parathyroid hormone, leading to hypercalcemia. It may present with symptoms like constipation, abdominal pain, psychiatric complaints as well as signs of nephrolithiasis, and osteoporosis that may need surgical treatment. PHPT is often underdiagnosed and undertreated. Our study aimed to review hypercalcemia in a single center to evaluate for undiagnosed PHPT.

Methods

A group of 546 patients from Southwest Virginia was selected using the EMR Epic (Epic Systems, Verona, USA)^ ^who had a diagnosis of hypercalcemia in the previous six months. Charts were manually reviewed, and patients were excluded based on the lack of hypercalcemia as well as previous testing of parathyroid hormone (PTH) levels. One hundred and fifty patients were excluded because of a lack of documented hypercalcemia. Letters were sent to the patients advising the patients to discuss whether a PTH would be indicated with their primary care provider (PCP). These patients’ charts were re-examined six months later and a determination of whether a PTH level was performed as well as any referrals explicitly for hypercalcemia or PHPT was done.

Results

A total of 20 (5.1%) patients had a new PTH test during the time assessed. Of these patients, five received referrals to surgical treatment and six received referrals to endocrinology for treatment (no patients received referrals to both).

Of those who obtained a PTH level, 50% of them had significantly elevated PTH levels consistent with the diagnosis of primary hyperparathyroidism. An additional 45% had levels of parathyroid hormone that were in the normal range but or most likely inappropriately normal for the concurrent level of calcium. Only one patient (5%) had a suppressed PTH level.

Conclusion

The impact of an intervention on clinician evaluation and treatment of patients with hypercalcemia has been previously tested and shown to be beneficial. In this study, the method of directly sending a letter to patients yielded clinically significant results, with 20 patients out of 396 (5.1%) having a PTH level tested. A majority had an overt or suspected parathyroid disease, and out of them, 11 underwent referral for treatment.

## Introduction

Primary hyperparathyroidism (PHPT) is a disorder in which one or more parathyroid glands overproduce parathyroid hormone. Common causes include parathyroid adenoma or parathyroid hyperplasia [1]. This overproduction leads to hypercalcemia as the most direct result and results in symptoms of abdominal pain and constipation, in addition to multiple other sequelae. Among the most severe of these are nephrolithiasis and osteoporosis with resultant vertebral and femoral fractures [2, 3]. Other consequences include accelerated functional decline in older patients [4]. In many cases, parathyroidectomy, the definitive cure, may be indicated [5]. Despite this, multiple studies have shown that PHPT is often underdiagnosed and thus undertreated [6-9]. In light of this, our study aimed to locate a population with hypercalcemia and undiagnosed PHPT and assess the effects of a direct message to the patient as an intervention on diagnosis and treatment rates.

## Materials and methods

Prior to the initiation of the study, the Carilion Health System (located in Southwest Virginia) institutional review board (IRB) approval was obtained. Once approved (IRB approval number #IRB-21-1463) a query of the electronic medical record (EMR) EpicTM (Epic Systems, Verona, USA), as well as augmentation with TrinetX (TrinetX, Cambridge, USA), was performed with a goal of finding outpatients with at least one elevated serum calcium (serum calcium ≥ 11.0 mg/dL) and no parathyroid hormone (PTH) value found. Other parameters on the population included age greater than 18 and the years when examined between 2021 and 2022. Although multiple options exist and vary regarding what normal, elevated, and low PTH levels are, in this study the decision was made to choose a PTH of 65 pg/mL as the cut-off point for elevation and 21 pg/mL as the cut-off point for depression - both based on previous studies and on local institutional cut-offs [6].

In total, a group of 546 patients were found that had elevated serum calcium and no PTH based on a computerized query. Charts were then manually reviewed by members of the research team to confirm query findings, and patients were excluded based on the lack of hypercalcemia (serum calcium ≥ 11.0 mg/dL) as well as previous testing of parathyroid hormone (PTH) levels. A total of 150 patients were excluded and 396 were included in this study.

Letters were sent to the 396 patients advising the patients to discuss whether PTH testing would be indicated with their primary care provider (PCP). A repeat query was run following the letters, six months later, and a total of 20 patients were found to have new reported PTH levels tested in the time period following the letters. These patients’ charts were re-examined manually and PTH values were verified. Additionally, any referrals over the same time frame (explicitly for hypercalcemia or PHPT) were also recorded.

## Results

Of the 396 patients initially included, all were found to have an elevated serum calcium (≥ 11.0 mg/dL) and no evidence of PTH value during the study period. After the letter was then sent, further inquiry was made into PTH testing, referrals to endocrinology, and referrals to surgery.

**Figure 1:**
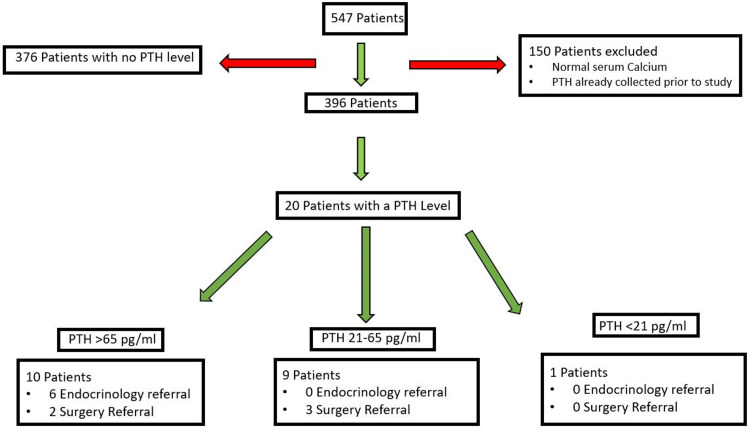
Screening, Randomization, and Follow-up of the Participants. PTH: Parathyroid Hormone PTH levels (for normal calcium): 21-65 pg/mL

A total of 20 (5.1%) patients (Figure 1) had a new PTH test during the time assessed. Of these patients, five received referrals to surgery treatment and six received referrals to endocrinology for treatment (no patients received referrals to both).

Of those who obtained a PTH level, 50% had significantly elevated PTH levels consistent with the diagnosis of primary hyperparathyroidism. An additional 45% had levels of parathyroid hormone that were in the normal range, most likely inappropriately normal for the concomitant level of calcium, consistent with a possible parathyroid disease as well. Only one patient (5%) had a suppressed PTH level. Stratification of the PTH levels tested is shown in Figure 1.

In patients with a PTH level >65 pg/mL, 80% (8 of 10) received a referral to endocrinology or surgery for the purpose of PHPT treatment. In patients with a PTH level of 21-65 pg/mL, 33% (3 of 9) were referred to surgery and no patients were referred to endocrinology for treatment. Lastly, in patients with a PTH level of less than 21 pg/mL, no referrals were made for treatment. Overall, 55% (11/20) of the patients who underwent PTH measurement received a specialist referral.

## Discussion

Hyperparathyroidism is a relatively common disorder that is seen in clinical practice [10-12]. It is generally diagnosed with a combination of elevated calcium and inappropriate parathyroid hormone level. Obtaining a PTH level in the face of hypercalcemia is highly valuable in the diagnosis of primary hyperparathyroidism [11]. Multiple options exist and vary regarding what normal, elevated, and low PTH levels are [4, 5, 13].

Like other previously conducted studies on this matter, we demonstrated that patients with elevated calcium levels are not always fully evaluated and treated for primary hyperparathyroidism [6-9].

Multiple studies have found that PHPT is underdiagnosed by a significant magnitude [7, 14, 15]. Balentine, et al. reported finding that 71% of patients expected to have PHTH based on their calcium levels never had a PTH tested and an additional 80% of those whose PTH was tested did not receive a surgical referral [7].

Previous studies have reported the use of a clinical decision-making tool within the electronic medical record (EMR) to aid the decision-making of physicians or advanced care practitioners (ACP) in patients with elevated calcium. This showed significant initial success, with a profound six-fold increase in the PTH level tested following the initiation of the tool [10]. The intervention method in this study differed as our goal was to enhance patient engagement by reaching out to patients directly while also making the letter visible to the patients’ PCPs.

Of the 5.1% of the 396 patients that met inclusion criteria and had a PTH level measured during the follow-up period, 19 (95%) had levels that were either elevated (PTH > 65 pg/mL) or inappropriately normal (21-65 pg/mL), indicating a high likelihood of PHPT. Rates of referral within patient groups can be compared to the Ballentin, et al. study, in which 21-29% of patients with a PTH > 65 pg/mL were referred to surgery, as this study showed a 20% rate of surgical referral, although notably this study also assessed endocrinology referrals which were not assessed in the Ballentine, et al. study (Figure 1) [7]. The tendency for a highly elevated PTH to lead to a specialist referral was somewhat less as compared to other studies [6, 7]. Limitations of this study include the small sample size and the fact that it is from a single center.

## Conclusions

This study confirms the results of prior studies, confirming that parathyroid hormone testing is not always done in patients with elevated calcium levels. This may result in a delay in diagnosis and treatment leading to adverse patient outcomes. The impact of an intervention on clinician evaluation and treatment of patients with hypercalcemia has been previously tested and shown to be beneficial. In this study, the method of directly sending a letter to patients yielded clinically significant results, with 20 patients out of 396 (5.1%) having a PTH level tested. A majority had an overt or suspected parathyroid disease and 11 (55%) of those were referred for treatment. Although this number of patients is small, the intervention did result in a large percentage of patients with a previously undiagnosed disease being identified. As physicians attempt to engage patients in healthcare decision-making, it may be helpful to quantify the effect of this on a larger scale in the years ahead.
